# Dental Antiquities

**Published:** 1859-04

**Authors:** 


*Dental Antiquities.*—The following curious dental advertisements were taken from the Maryland Journal and Baltimore Advertiser, for 1783 and '84, respectively. We are inclined to think that Mr. Snowden was the first dentist in Baltimore.—Eds.

Mr. Snowden, *Surgeon Dentist,* wishing to act on the most liberal principles, will give his advise *gratis* in all cases relative to the teeth and gums, either at his own apartments, Mr. Augustine's, in Calvert street, or at people's respective homes; and if any operation or attendance is deemed necessary, if the party is not perfectly satisfied, with pleasure will return his fee; he continues to scale and clean the teeth (in his much approved method and by a mode peculiar to himself;) he does not give the least pain—no roughness or disagreeable sensation attends his operation, and he seriously advises every person whose teeth are in the least foul or gums in a bad state, to have it performed, as the only possible means of preserving their teeth, which it will do to old age; he is happy that having attended a great number of respectable people, every individual has expressed the utmost satisfaction and content. The public have now an opportunity of preserving their teeth; he would wish to speak plain and be understood; he supplies the loss of teeth with the enamel or not, to answer every purpose of speech, &c., and look as well as those lost. Teeth that are uneven or displeasing, he renders even and agreeable, stops decayed teeth, preventing their further decay, and tooth-ache, and often can remove the decayed part entirely Children have many complaints of their teeth which parents ought to be attentive to, and which he can remove. Performs even other curious and useful operations. At a considerable expense he studied the Surgeon Dentist's art in Europe, and is regularly educated in physic and surgery, and will be happy to give the public entire satisfaction.

*July* 26, 1783.

Doctor Fendall having just arrived in town, has taken up his residence, for a few weeks, at Miss Young's, in Calvert street. It is not solely by lucre, or pecuniary views, the Doctor is prompted to offer his assistance to the public; he is urged by a desire to serve them; kind fortune has made him independent, and placed him far above the hungry tooth of want. To serve his country is his design, and to remove the vast variety of disorders the teeth, &c. are exposed to: These are matters of universal concern, and, he thinks, essentially require the attention of the public, though he is sorry to say, too slightly regarded by many. The teeth are often attended with complaints, which injure the whole system; deformities of them are frequeutly seen in common life, to be prodigiously injurious. The orator in the pulpit, or at the bar, will cut but an indifferent figure with loss of teeth; the private gentleman and the fine lady suffer no disadvantage, in social life, from a mouth full of teeth, white, regular and polished as ivory. How a deformity, in this particular, caricatures the appearance of many, amiable in every other feature, whilst beautiful and elegant teeth make some countenances agreeable, which have little else to recommend them.

Dr. Fendall cures the most inveterate scurvy (as it is vulgarly termed) in the gums, by carefully scaling from the teeth that corrosive tartarous substance, which impedes the gums from growing, infects the breath, and is a principal cause of the scurvy. This, if not timely prevented, eats away the gums, so that many people's teeth fall out sound. He prevents teeth from rotting, such as are decayed he keeps from becoming worse, even to old age; makes the gums grow firm to the teeth, and renders them white and beautiful; fills them with gold or lead, those that are hollow, so as to render them useful, and to prevent the air from getting into them and aggravating the pain; he transplants natural teeth from one person to another, which will be as firm in the jaw (without any ligament) as if they originally grew there; he engrafts natural teeth upon old stumps, and makes and fixes artificial teeth with the greatest exactness and nicety, without pain, so that the patient may eat, drink and sleep with them in the mouth as natural ones, from which they cannot be distinguished by the sharpest eye. When he fixes in artificial teeth, if not more than four, he gives them an enamel, which will preserve its whiteness by care, and entirely retain it. This method of fixing teeth is different now from all other methods. His antiscorbutic tincture (the peculiar virtues of which are well known in this town) he still recommends; it is greatly superior, in elegance and efficiency, to anything hitherto made use of for the teeth and gums; it is free from any corrosive preparation, will restore the gums to their pristine state, prevent the tooth-ache, and render the breath delicately sweet (if the tartarous substances are taken off by a skillful operator) and will remedy all the disorders which are consequence of scorbutic gums. Every person's may not want scaling—to those happy few, if they mean to keep them so, he recommends the dentifrice as the only rational method to preserve the beauty and goodness of their teeth and gums. The Doctor, having confined himself to that part of surgery which concerns the dentist's art, conceives he has not needlessly employed his time, especially when it is considered how extremely beneficial this art is to mankind. He begs attention of the public, and particularly of those who have children affected with disorders and deformities of the teeth. The teeth and gums are liable to the following complaints: various kinds of pains, proceeding from different causes; defluctions falling on the gums; obstructions and inflammations of the nerves and vascular parts of the teeth; acrid matter generated in the neighborhood of the teeth; fungous excresences and ulcers of the gums; (he has a case wherein he extirpated an excrescence, in the mouth of a young lady, as large as an Indian walnut) recess of the gums; exposure of the roots of the teeth; tartar of the teeth; and the recess of the gums occasioned by the tartarous concretions long neglected, attended with bad consequences destructive to the teeth and organs, and brings on inflammation. The discolored enamel, he fears, is too often mistaken for tartar. Looseness of the teeth, change of position, protrusion, total luxation, fractures of the alveolar parts of the jaw bone, of sharp splinters, portions of the teeth left behind in extraction, bruises and lacerations of the gums; matter collected in the sinuses, and often are cancelated substances of the lower jaw, caries and exostosis of the bones which form the sockets of the lower jaw. A singular case and a cure of his own, where a lady lost the coronoid and condyloid processes, with a large portion of the lower jaw, by a caries in the tooth—various kinds of caries on roots in the teeth: looseness, softness, bleeding of the gums, bad-smelling breath, and loss of palate. What the Doctor has said, he strongly hopes the impartial public will readily admit as a candid declaration of the truth, and the language of a man who would not, from any inducement, deceive them: undissembled truth, he thinks, should ever be predominent amongst mankind. Should the ladies and gentlemen find no relief, or be disappointed in their most sanguine expectations, from his operations, he will as readily return the money as he received it. He will wait on gentlemen and ladies in town, still charging no more than if waited on by them; if sent for four or five miles into the country where there are that number of patients, his price will be the same as in town. After the Doctor leaves this town, he intends visiting Annapolis, on his return home.

*Baltimore, July* 20, 1784.

